# Cassava brown streak disease and the sustainability of a clean seed system

**DOI:** 10.1111/ppa.12453

**Published:** 2015-09-21

**Authors:** C. F. McQuaid, P. Sseruwagi, A. Pariyo, F. van den Bosch

**Affiliations:** ^1^ Rothamsted ResearchWest CommonHarpendenAL5 2JQUK; ^2^ Mikocheni Agricultural Research InstitutePO Box 6226Dar es SalaamTanzania; ^3^ National Crops Resources Research InstitutePO Box 7084KampalaUganda

**Keywords:** cassava model, disease management, planting material

## Abstract

One method of reducing disease in crops is the dissemination of disease‐free planting material from a multiplication site to growers. This study assesses the validity and sustainability of this method for cassava brown streak disease, a threat to cassava crops across East Africa. Using mathematical modelling, the effects of different environmental and control conditions on pathogen spread were determined in a single‐field multiplication site. High disease pressure, through large vector populations and disease in the surrounding area, combined with poor roguing practice, resulted in unsuccessful disease suppression. However, fields may produce sufficiently clean material for replanting if these factors can be overcome. Assessing the sustainability of a low‐pressure system over multiple harvests, well‐managed fields were found to maintain low disease levels, although producing sufficient cuttings may prove challenging. Replanting fields from the previous harvest does not lead to degeneration of planting material, only cutting numbers, and the importation of new clean material is not necessarily required. It is recommended that multiplication sites are only established in areas of low disease pressure and vector population density, and the importance of training in field management is emphasized. Cultivars displaying strong foliar symptoms are to be encouraged, as these allow for effective roguing, resulting in negative selection against the disease and reducing its spread. Finally, efforts to increase plant multiplication rates, the number of cuttings that can be obtained from each plant, have a significant impact on the sustainability of sites, as this represents the primary limiting factor to success.

## Introduction

Cassava brown streak disease (CBSD), caused by *Cassava brown streak virus* (CBSV) and *Ugandan cassava brown streak virus* (family *Potyviridae*, genus *Ipomovirus*), is a serious constraint of cassava crop production for farmers and growers throughout East Africa (Legg *et al*., 2011). Due to its historically narrow geographic range compared to another key cassava disease, cassava mosaic disease (CMD), CBSD has not received much attention until recent years (Hillocks & Jennings, 2003). Indeed, it was not until the last decade that a whitefly vector (*Bemisia tabaci*) was even reported to semipersistently transmit the disease (Maruthi *et al*., 2005; Legg *et al*., 2011), and there is still potential for other, currently unknown, vectors to exist. Despite this lack of attention, or perhaps as a result of it, CBSD is thought to be one of the most economically important viral diseases affecting cassava in Africa (Muhanna & Mtunda, 2003; Patil *et al*., 2015). With the widespread prevalence of CBSD throughout coastal and, more recently, parts of inland East Africa, this represents a severe constraint on production leading to significant reductions in yield (Nichols, 1950; Bock, 1994; Legg *et al*., 2011). A general review of CBSD can be found in Hillocks & Jennings (2003), with more recent knowledge on its epidemiology in Legg *et al*. (2011).

Many different management strategies have been both proposed and imposed in order to control cassava diseases, including the use of resistant varieties, disease‐free (‘clean’) planting material and the removal of infected plants (‘roguing’), although these strategies have met with mixed success (Hillocks & Jennings, 2003). For CBSD, few resistant or tolerant cassava varieties currently exist (although see Kanju *et al*., 2003). In addition, none of the known resistant varieties are resistant to both causal viruses (Legg *et al*., 2011). To make matters worse, one of the primary CBSD‐resistant varieties used in Tanzania is itself highly susceptible to CMD (Kanju *et al*., 2003). Given that CMD is often a problem in areas affected by CBSD, the use of resistant varieties alone is likely to be insufficient to prevent the spread of multiple cassava viral diseases. This is made even more difficult by the fact that resistant varieties are unlikely to be adopted in the absence of disease unless they are preferred by growers for other additional aspects, such as their taste. Controls such as the application of insecticides or roguing are also unpopular due to the unacceptable increase in costs for subsistence farmers (Legg *et al*., 2014), as well as the difficulty in successfully identifying diseased plants. Roguing for CBSD is complicated by both the subtle disease symptoms of CBSD and the variability in symptom expression of the disease between cassava varieties, and even plants, making disease detection difficult for the untrained (Legg *et al*., 2011; Rwegasira & Rey, 2012; Patil *et al*., 2015).

One alternative method of control (Kanju *et al*., 2003; Legg *et al*., 2011) includes the growing and roguing (by trained practitioners) of plants in isolated fields located in areas of low ‘disease pressure’ (here, this is considered to relate to the primary infection rate affecting the field, although it is also used elsewhere to refer to the vector density or the presence of diseases in an area). The isolation of these clean propagation fields helps to slow the arrival of disease (Legg *et al*., 2011), while roguing has previously been proven to be effective at ridding an area of CBSD (Jameson, 1964; Hillocks & Jennings, 2003), possibly due to the low retention time of the causal viruses by infected whitefly (Legg *et al*., 2011). Cuttings from these fields are then distributed to local growers. It is thought that the distribution of this clean planting material, often referred to as clean ‘seed’, will reduce disease pressure in communities by ensuring that the majority of crops are, at least initially, relatively disease‐free (Kanju *et al*., 2003). In comparison, growers in affected areas that replant fields with cuttings traded with neighbours or taken from their own fields often perpetuate disease, maintaining levels of infection for CBSD of around 30%, occasionally even exceeding 50% (Rwegasira *et al*., 2011; Mbewe *et al*., 2015; Patil *et al*., 2015).

The dissemination of this clean planting material naturally requires an entire production chain. Plant breeders and researchers develop potentially tolerant or resistant seed, operating sites that receive, screen and propagate pre‐basic (breeder) and basic (foundation) seed. This is distributed to seed agencies and private seed companies that continue to propagate the virus‐tested material, before further distribution at a regional level. Here it may be sold to individual growers or cassava seed entrepreneurs, who multiply and sell quality‐declared planting materials to local growers in their community as described above. In the last two stages of this process, seed certification agencies act to assure the quality of the planting material, through virus testing and certification.

Clean seed systems are currently in the process of being established in both Tanzania and Uganda, and the work presented here aims to offer guidance for these. It is the viability and sustainability of the sites managed by cassava seed entrepreneurs (tertiary multiplication sites) that this study focuses on in particular. The focus on these sites determines the infection levels that is expected in the system, as well as guidelines for both the required quantity and quality of planting material produced. Many different factors will affect the planting material in these fields (Thresh & Cooter, 2005); yet no experimental work currently exists to support their implementation. In this case, modelling to simulate the spread of the pathogen and its effect on disease incidence levels in cuttings represents a useful tool to look at potential outcomes for the system. Such a model is created here. While no data are currently available to validate the model, by basing it on reasonable assumptions, different control schemes can be tested under different disease scenarios at a minimum cost in both time and money. Once data may be obtained from any seed systems, this data can be used to feed back into the model to improve its accuracy. Similar approaches have been used in a range of other pathosystems and are well validated (Gilligan & van den Bosch, 2008; Parnell *et al*., 2009).

The model presented here investigates the spread of the pathogen within an individual multiplication site field in order to determine the percentage of infected cuttings amongst those that are distributed to growers. To investigate the viability, it was determined whether the site can produce enough planting material that has levels of disease within a given set of guidelines. To test the sustainability, the degeneration rate of the planting material produced was determined, i.e. the rate at which disease in the system increases over multiple growing seasons if each subsequent crop is replanted from the previous harvest. If the crop is replanted from cuttings of the previous crop, then its initial incidence will be determined by that in the previous crop, potentially leading to higher levels of infection at each replanting. However, the use of cuttings collected from the same field at the previous harvest may be necessary to reduce costs, as the purchase of new clean planting material from higher up the value chain at every planting could prove expensive. Information on the rate of degeneration is therefore key for decision‐making, indicating when a grower should stop recycling planting material and pay for a new set. It may even be that, if positive selection identifying disease‐free plants is efficient, the option of replanting from previous harvests could be nearly as effective as purchasing tissue‐culture derived planting material independently, making it a cheap, sustainable alternative.

Here, the model is used to compare the effect of varying parameters, such as disease pressure, whitefly population, initial infection levels and the quality and frequency of roguing, on the incidence of disease amongst the cuttings distributed to growers after a harvest. The model may then be used by researchers on the ground to suggest guidelines to a range of people, from extension agents to crop inspectors and even seed producers, to inform decisions on different strategies to increase success rates or to decrease costs under a given set of circumstances. Unless strict management of fields is in place, they may quickly be rendered both nonviable and unsustainable, despite their relative isolation. A key challenge lies in the number of plants that are available after roguing to provide cuttings at the end of a season, which represents a severe constraint on production. However, if this can be overcome then the system may be successfully implemented, increasing access to clean planting materials for growers.

## Materials and methods

### Outline

A stochastic, individual‐based model was constructed, using discrete time‐steps for the spread of CBSD in a field of cassava plants (Gibson, 1997; Keeling & Rohani, 2008; Sisterson & Stenger, 2013). The plants are susceptible to infection by CBSV from both internal and external sources, and are divided into four classes; susceptible, latent, infectious and removed. Plants in each class are affected by different infection‐related processes, where a number of events may occur in each time‐step of 1 day, including periodic harvesting, roguing (if successfully identified as infected) and infection‐related events. Susceptible plants may become latently infected, latently infected plants progress to an infectious state where they infect other plants, and, finally, infectious plants remain infectious indefinitely unless removed during a roguing or harvesting event.

Each plant is considered separately, calculating the probability of an event occurring for that plant. Events that occur stochastically are then picked according to these probabilities. Each plant's status is then updated to reflect the events that have occurred, before continuing to the following day and repeating. This continues until the growing season is complete, at which point the remaining plants are harvested. A total of 10 000 cassava plants are all planted at the same time in a 100 × 100 m field, with a 1 × 1 m spacing between plants and rows. Growing periods for cassava can be highly variable: between 7 months and 2 years. Here it is presumed that the growing period is 1 year, as is likely to be the case for tertiary multiplication sites.

An outline of the model is given below, which is simulated using the C programming language. A full list of further epidemiological parameters used in the model, and their values, can be found in Table 1, and details of the model derivation can be found in the Appendix S1.

**Table 1:** Model parameters and values

Parameter	Description	Value	Value range	Units	Reference
*λ*	Primary infection rate	0·9 × 10^−6^	0–1·8 × 10^−5^	Plants day^−1^	Fargette *et al*. (1990), Legg (1994), Holt *et al*. (1997), Jeger *et al*. (2004)
2/*α*	Mean dispersal distance	5·25	1–11·5	m	Fargette *et al*. (1990), Fargette & Vié (1995)
$\overset{¯}{\beta }$	Rate of spread	0·61	0·406–0·813	m day^−1^	Fargette *et al*. (1990), Fargette & Vié (1995)
*g*	Infection progression rate	0·033	0·018–0·048	Plants day^−1^	Maruthi *et al*. (2005), Mware *et al*. (2009), Mohammed (2012)
*P*	Probability of successful roguing	0·7	0–0·93	–	Rwegasira (2009), Abaca *et al*. (2012), Rwegasira & Rey (2012)
*h*	Reversion rate	0·25	0·05–0·45	–	Mohammed (2012)
*c*	Probability of successful selection	0·7	0–0·93	–	Rwegasira (2009), Abaca *et al*. (2012), Rwegasira & Rey (2012)
*v*_max_	Maximum whitefly population	–	5–110	Vectors plant^−1^	–
*i*	Initial infection incidence	3	0–5	%	J. P. Legg, IITA, Tanzania, personal communication

### Infection events

Cassava brown streak disease is spread in practice primarily through a whitefly vector, *Bemisia tabaci*, as well as by the replanting of infected cuttings (Storey, 1936; Maruthi *et al*., 2005). Infection through the vector in this model occurs due to either primary infection, where infected whiteflies from an outside source enter the field and infect a plant, or secondary infection, where whiteflies transmit the pathogen from an infectious plant to a susceptible plant within the same field. Rainfall and wind conditions, which may affect whitefly movement into a field (Legg, 1994), are implicitly incorporated into the whitefly population parameters and are assumed to be constant here, although this may be varied.

In each time‐step of 1 day, the probability of a plant *x* becoming infected is therefore determined by the rate of spread of the pathogen from infected sources within the field (at rate $\overset{¯}{\beta }$ over mean distance 2/*α*, where the norm ¦¦·¦¦ measures the distance between two plants) as well as the movement of infected whitefly into the field (primary infection occurring at rate *λ*). See Appendix S1 for a derivation of this. In addition, the whitefly population changes with crop age *t* according to *v*(*t*), where

$$v\left(t\right)=v_{\max}\left(-\frac{1}{2}\text{cos}\left(\frac{2\pi t}{365}\right)+\frac{1}{2}\right),$$

for maximum vector population *v*_max_, reaching a peak 6 months after the crop was planted. Susceptibility of an individual plant changes with age *τ* according to *a*(*τ*), where

$$a\left(\tau \right)=3\cdot 01\left(\tau -1\cdot 07\right)e^{-1\cdot 37(\tau -1\cdot 07)}+0\cdot 23,$$

for *τ *> 1 and *a*(*τ*) = 0 otherwise, initially peaking 2 months after planting before declining rapidly to an equilibrium (Fargette & Vié, 1994, 1995). For further details, see Appendix S1. This gives, for each plant *x*, a probability of infection in 1 day given by

$$P\left(x\right)=1-e^{-v(t)a(\tau )z},$$

where the rate of infection is given by

$$z=\frac{\overset{¯}{\beta }\alpha^{2}}{2\pi }\left(\sum_{y\in infected}e^{-\alpha ||y-x||}\right)+\lambda$$

for infectious plants *y*. For each plant *x*, the total probability of infection is therefore calculated by summing the rates of infection from nearby infectious plants, as well as the rate of infection from outside the field. Plants then progress from a latently infected to infectious state at rate *g*.

### Roguing and harvesting

Plants move to the removed class through periodic roguing and harvesting events, which occur at regular frequencies. First, consider roguing (with probability of success given by *P*), which occurs after a variable interval (*q*) within the first 2 months after planting, and double that interval thereafter. It is unclear whether roguing will promote dispersal of the whitefly, but as no data for this currently exist, the possibility is not included here. If roguing occurs within the first 2 months of planting, a replacement cutting is planted (Dr K. Mtunda, NARI Tanzania, personal communication), but thereafter no replacement occurs (see Sisterson & Stenger, 2013) for the effects of replacement on disease management). Note that only infectious plants display symptoms (it is assumed the disease is similar to CMD in this aspect, where symptom expression is strongly correlated to virus content, see Fargette *et al*., 1987), and must be correctly identified as such in order to be rogued. Although symptoms appear most strongly in plant roots for CBSD, which cannot be evaluated before harvest, the correlation coefficient between foliar disease symptom severity and root disease symptom severity is high, implying that plant leaves are still a useful tool for determining disease presence or absence (Abaca *et al*., 2012).

After a growing season of 365 days, the remaining plants are harvested and cuttings selected for distribution. From the harvested crop, a new crop of cassava is also planted. The proportion of infected plants determines the number of infected cuttings harvested and replanted, and hence the number of latently infected plants in the new crop. However, reversion (*h*, the percentage of healthy plants that are produced from cuttings of infected plants) and cutting selection (*c*, the probability of detecting infected cuttings) reduce this number. Both these two factors are unlikely to affect an individual plant, as plants that are prone to reversion often display less symptoms, reducing the effect of cutting selection (Fargette & Vié, 1995).

Due to the current lack of knowledge surrounding reliable dispersal parameters for CBSD, the following section considers the importance of uncertainty in these parameters for model results. The effect on disease incidence and viable cutting numbers of parameters that may potentially be varied, such as whitefly population numbers, roguing intervals and cutting selection, are subsequently investigated. Finally, the degeneration of planting material over time is modelled, as disease becomes established in the system.

## Results

### Model simulation of CBSD spread

The model focuses on a single, isolated field, which represents a clean seed system multiplication site; planting material obtained from breeders is multiplied here by entrepreneurs for sale to local growers. The important factors are the total number of cuttings available to distribute to growers, and the proportion of those cuttings that are infected, either latently or in a fully infectious state. The sustainability of the system is also investigated, to determine whether clean seed systems can be replanted from cuttings taken from within the fields at harvest, or whether the planting material will degenerate over a number of harvests.

**Figure 1:**
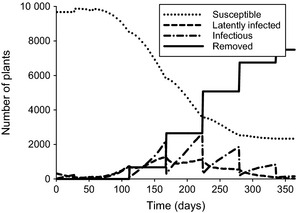
Plant population dynamics with time. Varying levels of infection in the population are considered as the disease spreads, in terms of number of individuals in a particular disease state. Periodic roguing causes sharp declines in the number of infectious plants, while replanting within the first 2 months may lead to an increase in the number of susceptible plants. The final population sizes at time *t *=* *365 days are used to calculate the disease incidence and number of available cuttings in the field.

As an example, Figure 1 describes a simulation of the plant population dynamics over a 1‐year‐long growing season for a given set of parameters. Disease parameters, namely *α*, $\overset{¯}{\beta }$, *λ*,* g* and *h*, are chosen uniformly from the ranges described in Table 1. Field parameters are given by *v*_max_ = 55 whitefly per plant, *i *=* *3%, *P *=* *90% and *q *=* *28 days. Note that by the end of the season a large proportion of the crop, nearly three‐quarters, has been removed due to infection and subsequent roguing.

The simulation enables the disease incidence in the crop at the end of the season to be determined, as well as the total number of cuttings available. Repeating the simulations a number of times, the environmental stochasticity is much greater than the demographic stochasticity, which has little effect on the results. The environmental and disease parameters are therefore resampled at every run of the model in order to obtain an average incidence and number of cuttings for a given set of field parameters. These averages from the end of each season are used to construct the remaining figures in this section.

### Variation of disease parameters

**Figure 2:**
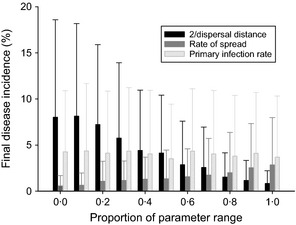
Comparisons of the effects of parameter ranges for dispersal distance (*α*), rate of spread ($\overset{¯}{\beta }$) and primary infection rate (*λ*) on final disease incidence in a field with a high (80–110 individuals per plant) whitefly population. The *x*‐axis measures the value for the appropriate parameter as a proportion of the total range found in Table 1. All other parameters are chosen from a uniform distribution given in Table 1. The bar gives the mean and the whiskers one standard deviation for 300 runs of the model.

The importance of the range of some key disease parameters is investigated, establishing an idea of the sensitivity found in the model results. In Figure 2 one infection parameter at a time is varied, and the others are chosen uniformly (to identify the robustness of the model to outlying parameters) at random from the ranges given in Table 1, which are taken from the literature if present or are calculated in Appendix S1. The effect of varying the primary infection rate *λ* is very low at such low levels, where the amount of primary infection entering a field is negligible, and makes very little difference to the final infection incidence. However, as the range increases to medium or high disease pressure, the effect becomes more apparent. On the other hand, varying the mean dispersal distance 2/*α* can significantly decrease infection incidence, as can decreasing the rate of spread $\overset{¯}{\beta }$ to a lesser extent. As these parameters are currently unknown for CBSD, this information gives an idea of the uncertainty in the results below, which may be of one order of magnitude for particularly extreme values of *α* and $\overset{¯}{\beta }$. Results are similar for a low (5–35 individuals per plant) whitefly population (Appendix S2). Similarly to primary infection, cutting selection after harvest makes no noticeable difference to levels of incidence and number of plants available for multiplication (Appendix S2).

### Variation of field parameters

**Figure 3:**
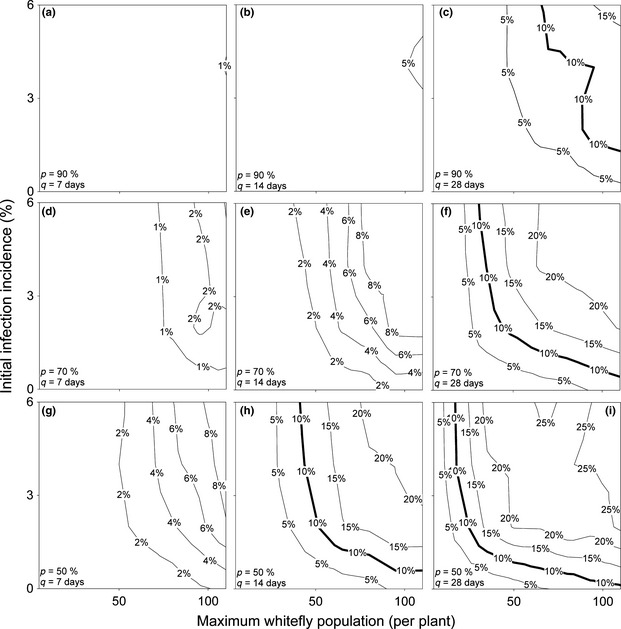
Mean final incidence of disease (%) in cuttings distributed to growers at harvest over a range of parameters for a low disease pressure system. For each plot, *x*‐axis is the maximum whitefly numbers (per plant) and *y*‐axis the size of an initial source of infection (%). Roguing interval varies across columns, where the interval (*q*) given below is for the first 2 months after planting, after which the interval doubles. Roguing occurs weekly in column 1 (plots a, d, g), fortnightly in column 2 (plots b, e, h) and monthly in column 3 (plots c, f, i). Roguing success, or the probability of successfully detecting a plant with symptoms (*P*), varies across rows, and is 90% in row 1 (plots a–c), 70% in row 2 (plots d–f) and 50% in row 3 (plots g–i). Bold lines represent the guideline maximum infection level of 10%, as suggested by the 5CP project, in the cuttings Dr J. P. Legg, IITA, Tanzania, personal communication.

**Figure 4:**
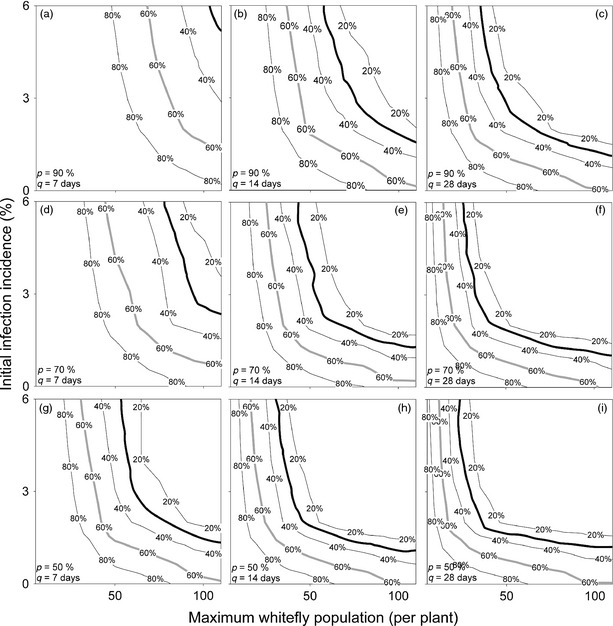
Mean number of plants available at harvest to provide cuttings (as a percentage of the initial plant population) to distribute to growers, considered over a range of parameters for a low disease pressure system. *x*‐axes, *y*‐axes, columns and rows vary as described in Figure 3. Bold grey lines represents the minimum percentage of plants remaining in a 5 acre field at harvest to distribute sufficient cuttings to plant 30 acres of fields if multiplication rates are low (10‐fold), while bold black lines, at 30% of the population, represent the same for high multiplication rates (20‐fold).

Field parameters, namely *v*_max_, *i*,* P* and *q*, are varied and the incidence (Fig. 3) and plant population size as a percentage of the original population (Fig. 4, which is used to calculate the number of cuttings available) are calculated. For example, multiple simulations such as that shown in Figure 1 are run for a given combination of field parameters. From this the average final incidence and population size is calculated, which determines the value at one point each in Figures 3 and 4, respectively. In this case, the field parameters used in Figure 1 correspond to the set of parameters used to calculate the incidence and population size at the centre point of the top right‐hand plot in Figures 3 and 4, respectively. These results can then be used to identify how the field parameters can be controlled to increase the probability of a clean seed system being successful. Further details on the effect of disease pressure on this can be found in Appendix S2.

### Disease incidence

The incidence of disease in cuttings distributed to growers may be kept low for certain parameter combinations considered here (see Fig. 3). This is below the 10% guidelines proposed, and currently being appended to the Seed Act of Tanzania, by the 5CP project in conjunction with the Tanzania Official Seed Certification Institute (Dr J. P. Legg, IITA Tanzania, personal communication). Indeed, of the four factors affecting the infectious population at harvest (low whitefly populations, low initial infection incidence, frequent and effective roguing), only two are required for the incidence to be kept at acceptable levels. It is through poor roguing or large numbers of whitefly that incidence becomes potentially problematic. It is only when roguing is conducted monthly, or fortnightly but with a roguing efficiency of less than 50%, that the final incidence of infection in plants distributed to growers exceeds 10%. Even this scenario may be avoided by reducing whitefly numbers to less than 50 whitefly per plant, although this may be an unfeasibly expensive solution. Practitioners should also be warned that if the whitefly population is high, then although roguing may remove the majority of plants with symptoms, symptomless or latently infected plants may still maintain the presence of disease in the field, resulting in cycles of increasing infection.

### Proportion of plants available for cuttings

A greater challenge may lie in the proportion of plants available for cuttings, which is reduced to less than 40% even for relatively low whitefly numbers and frequent, successful roguing (Fig. 4). This will have a significant impact on the commercial viability of the system, as it affects the number of growers that can replant with the clean planting material. Given that the rate of multiplication using stem cuttings is already slow (10‐ to 20‐fold (Guthrie, 1987)), this implies that production levels could easily drop to much lower levels (4‐ to 8‐fold of the original number of cuttings) unless the disease is carefully managed. For a 5‐acre field clean seed system, required to distribute planting material to 20 growers with 1 acre fields each, 30 acres worth of cuttings are required. If the multiplication rate is 10‐fold, this implies that to produce sufficient cuttings 60% of the initial number of plants are required at harvest, while if the multiplication rate is 20‐fold, only 30% of the initial plants are required. It is clear that, in either case, this is a much stronger constraint than the disease incidence in the cuttings (see bold lines in Fig. 3 versus Fig. 4).

### Changes over multiple seasons

**Figure 5:**
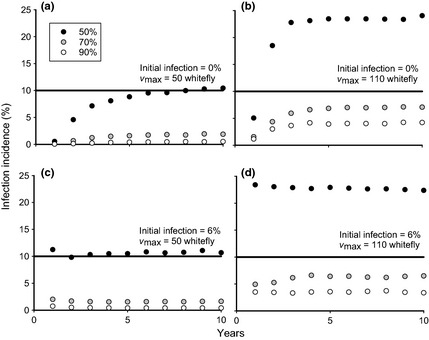
Degeneration of the planting material over a 10 year period. The *y*‐axes measure the percentage of infected cuttings at the beginning of each planting cycle, while for each set of symbols in each plot the roguing success rate varies as described in the key. The maximum number of whitefly per plant is either 50 (plots a, c) or 110 (plots b, d). The infection level in the initial cuttings planted for the first season is either 0% (plots a, b) or 6% (plots c, d). Roguing occurs fortnightly for the first 2 months after planting and monthly thereafter, as is currently practised in the field. All other parameter ranges may be found in Table 1. Bold lines represents the guideline maximum infection level in the cuttings.

Changes in the disease incidence and proportion of plants available for cuttings over multiple seasons are investigated, where roguing frequency is set to be fortnightly for the first 2 months in a growing season and monthly thereafter, as in real systems (Dr K. Mtunda, NARI Tanzania, personal communication). Degeneration (increase in infection) of the planting material over growing seasons is assessed when different whitefly populations are present (Fig. 5), at different roguing success rates *P*. Each plot was averaged over 300 random systems, in order to investigate how the planting material degenerated over 10 growing seasons of a year each.

The infection frequency within the field does in fact equilibrate, which is perhaps a little unexpected, but is also encouraging. In general, for the parameters discussed in this example, a system will only distribute cuttings with more than 10% infection levels if roguing success is low. This has important implications, as it appears that clean seed systems, according to this model, may maintain infection levels within suggested guidelines.

Figure 5 can also be used to determine the different circumstances under which it would be best to reduce each of the initial infection, whitefly population and roguing success rate. For example, increasing the roguing success rate from 50 to 70% is more effective than reducing the whitefly population from 110 to 80 individuals per plant, no matter the levels of initial infection. This stresses the importance of breeding for foliar symptoms, which aid negative selection for growers, although scarce breeding resources might make this a low priority compared to breeding for resistance and yield. On the other hand, increasing the roguing success rate from 70 to 90% is about as effective as reducing the whitefly population as before.

**Figure 6:**
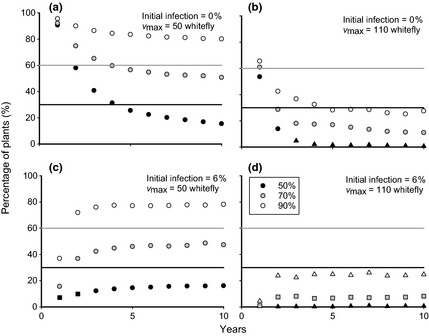
Percentage of plants available for cutting distribution at the end of each season over a 10 year period. For each set of symbols in each plot the roguing success rate varies as described in the key. Circles represent years in which sufficient cuttings are produced to replant the multiplication field itself, squares represent years in which the field could not be fully replanted if the multiplication rate was 10‐fold, and triangles represent years in which the field could not be fully replanted if the multiplication rate was 20‐fold. It is assumed that sufficient cuttings are always available for replanting during the decade‐long period. The maximum number of whitefly per plant and the infection level in the initial cuttings vary as in Figure 5, with the same set of parameter values and roguing at the same interval. Bold grey lines represents the minimum proportion of plants remaining in a 5 acre field at harvest to distribute sufficient cuttings to plant 30 acres of fields if multiplication rates are high (20‐fold), while bold black lines represent the same for low multiplication rates (10‐fold).

The effect that the degeneration described above has on the proportion of plants left in a field at the end of each season, which are used to distribute cuttings to growers, was then investigated (Fig. 6). It is assumed that there are sufficient plants to replant the field itself, which may not always be the case. Such alternative scenarios are marked with different symbols in Figure 6.

Only with both low whitefly populations and efficient roguing will as many as 50% of the initial plants be maintained for cutting distribution when roguing fortnightly; one control factor alone is insufficient. This imposes severe restrictions on the number of growers that may be supplied with clean planting material, and consistently falls below the threshold required to produce sufficient seeds even for high multiplication rates. Despite this, if, for example, a system could be planted that had 100% clean planting material, then it is possible that it could still be used to distribute a reasonably high number of cuttings for the first few years after planting, even if roguing was poor. However, this restriction to a case with no initial infection whatsoever does limit the approach somewhat, and it is observed that the systems reach an equilibrium fairly rapidly, making this only viable for a limited number of years. In conclusion, therefore, a clean seed system may not be sustainable in terms of cutting numbers, and hence may require planting material to be brought in from outside sources on a regular basis.

**Figure 7:**
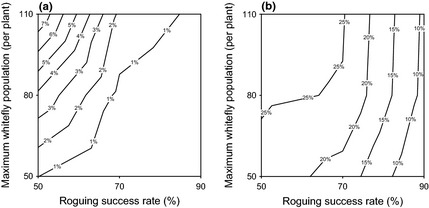
Equilibrium incidence levels over a 10‐year period if roguing is conducted (a) weekly or (b) monthly for varying roguing success rates and whitefly populations. Results are for final disease incidence percentage.

The incidence of disease in each system in Figures 5 and 6 stabilizes towards a noisy equilibrium over time, which is similar irrespective of the initial infection incidence. Results are qualitatively similar if roguing is conducted weekly or monthly, and the equilibrium values for 300 repetitions of the model at these roguing intervals with an initial incidence of 4% are shown in Figure 7. For similar results for a system in an area with medium levels of disease pressure, see Appendix S2.

Roguing must always occur frequently for a field to be sustainable; if this condition is ignored then incidence levels can climb, and, for all but the highest roguing success rates, quickly reach unacceptable levels. In this case, even the control of whitefly numbers to less than the 50 individuals per plant considered here may be insufficient to reduce the equilibrium disease levels. However, roguing weekly (Fig. 7a) does, in fact, maintain infection levels at sufficiently low levels to be considered sustainable, and has been recommended in the past (Kanju *et al*., 2003), although again high whitefly numbers may lead to the production of insufficient cuttings.

### Disease pressure

Many clean seed systems are currently based in areas of low disease pressure (Appendix S2; low values of *λ* equivalent to 0–2·5 new infections per fortnight in a field of 10 000 plants), and those systems are focused on here, as the reduction in number of plants available after a growing season in such systems is less restrictive than those with higher disease pressure. As the disease pressure increases, the initial infection level in fields has less of an effect, as disease caused by external sources becomes far more frequent than disease resulting from an initial infection (Appendix S2).

However, for particularly high disease pressure (given here by 2·8 × 10^−3^ < *λ *< 2·08, levels that may never be reached), the number of plants is so reduced by roguing, no matter how frequent or effective, that no more than 10% of plants remain to provide cuttings, and large proportions of these may be infected (Appendix S2). Establishment of multiplication sites in areas of high disease pressure would not be recommended, unless a particularly resistant variety of cassava could be planted, in which case the model could be used to determine the sustainability of that particular resistant variety. Even for a system under medium levels of disease pressure, whitefly numbers must be kept low, below 50 individuals per plant, to have any real hope of keeping the system sustainable.

## Discussion

As a practical example of putting the current findings to work, consider a scenario in which a grower owns a 5 acre field of disease‐free cassava, from which they wish to distribute cuttings to other members of a farmer group (numbering 25 members here), each of whom owns a 1 acre field. If the rate of multiplication is taken to be 10‐fold, then the grower requires one half of their plants to remain at harvest in order to supply sufficient planting material to the members of the group. It is assumed that the farm is located in an area of low disease pressure, and the seed company can guarantee that their initial planting material has 5% or less infection, while a maximum acceptable level of infection in the cuttings that they distribute is 10%. If there are 30 whitefly per plant in their field, then to obtain sufficient cuttings that have low enough levels of disease, they must either rogue weekly, or fortnightly but particularly effectively (with a success rate at identifying infected plants of 70% or higher). It may now be decided whether it is more effective to train the grower thoroughly in the identification of diseased plants, or to ensure that they rogue sufficiently frequently.

Although very little of the epidemiology of CBSD is known, the present model may be used to give an indication of the range of outcomes that might be expected from clean seed systems under different conditions. Further work on the epidemiology of CBSD, in particular the mean distance and rate of spread of the pathogen as well as the probability of roguing success, could then be used to increase the precision and accuracy of these results. Practically, the model indicates that clean seed systems can be maintained, and unless significant evidence to the contrary comes to light, it can be assumed that tertiary clean seed systems may be both viable and sustainable in areas of low disease pressure if roguing practice is of a sufficiently high standard and whitefly populations are kept low. In this case, the primary limiting factor of such systems will be the number of cuttings that are produced, which may fall below the total required to replant a field and deliver sufficient planting material to nearby growers.

The model results can be used as a guideline for the frequency and meticulousness of roguing that is required for a successful field under particular circumstances, as well as the pressure from whitefly populations above which intervention (through, for example, insecticides or increased roguing) is required or the field is best abandoned. However, it is worth noting that the establishment of an equilibrium infected population suggests that it may not be possible to rid a system of disease entirely.

In conclusion, the following advice is suggested for future practice and research. Clean seed systems appear to be viable primarily in areas with low disease pressure and few whitefly, suggesting that their establishment in higher disease‐pressure areas is unlikely to be successful. The training of growers, both in the identification of diseased plants and in careful field management through frequent roguing, is likely to significantly improve the success rate of the systems. In comparison, expending large amounts of resources on ensuring a complete lack of disease within the initial seed distributed to a clean seed system may not overly impact the sustainability of the system. Additionally, the breeding of cultivars to display clear foliar symptoms, which may accidentally be bred against, could in fact greatly aid negative selection for the disease, although more work on the link between symptom expression and virus titre is required here first. The increase in size of clean seed tertiary multiplication fields would lead to an increase in cutting numbers, removing this as a constraint, although this may be difficult to achieve for the average farmers' group. Finally, and most importantly, may be the increasing of multiplication rates for plants through appropriate grower training. This could help to remove the primary constraint of low cutting numbers on clean seed systems, making them more likely to be successful. Therefore, a clean seed system may be both a viable and a sustainable method with which to combat CBSD. However, both careful management and further research are required to ensure this.

## Supporting information


**Appendix S1.** Derivation of the model and parameters.

**Appendix S2.** Additional results.
