# Supplementary material for: Cassava brown streak disease and the sustainability of a clean seed system
Source: Plant Pathol. 2015 Sep 21;65(2):299–309. doi: 10.1111/ppa.12453 (PMC4950135; doi:10.1111/ppa.12453)
Supplement: Supplementary file 1 — Appendix S1. Derivation of the model and parameters. [file PPA-65-299-s001.pdf]

Appendix A. Model derivation

Infection rates for CBSD are largely unknown, although work is currently underway to address this deficit (Ms C. Gwandu, pers comm, 12th Nov 2013). We therefore model the spread of CBSD analogously to the spread of CMD, making assumptions where the diseases are known to differ.

I. Primary infection

We presume that the primary inoculation rate is constant for all susceptible plants within the field. This is calculated from the rate of infection with CMD of a plant host by an infected insect vector:

$$k = 0.008 \text{ vector}^{-1} \cdot \text{day}^{-1} \text{ (range 0.002-0.032)},$$

the number of vectors per plant:

$$m = 35 \text{ plant}^{-1} \text{ (range 5-500)},$$

and the proportion of vectors that are infected:

$$n_i = 0.01 \text{ (range 0.0018-0.13)},$$

where parameter values are taken from Fargette et al. (1990); Holt et al. (1997); Jeger et al. (2004) and Legg (1994). Note that the vector population is taken from Fargette et al. (1990) in particular, from which the secondary infection rate below is calculated. We therefore obtain primary infection rate

$$\lambda = kmn_i = 0.0028 \text{ plant}^{-1} \cdot \text{day}^{-1} \text{ (range 1.8x10}^{-5}\text{-2.08)}.$$

However, it is known that movement of CBSD inoculative vectors over distances of greater than 100m

between fields is uncommon (Dr F. Tairo, pers comm, 14th Nov 2013, see also Patil et al., 2014). Clean seed systems are separated from nearby fields by a distance of 100m or more, so using the above values for CMD infection we assume that the primary infection rate is given by $0 \leq \lambda < 1.8\text{x}10^{-5}$. Although the primary focus is low disease pressure systems, we also consider the effects of medium ($1.8\text{x}10^{-5} \leq \lambda < 2.8\text{x}10^{-3}$) and high ($2.8\text{x}10^{-3} \leq \lambda < 2.08$) disease pressure, although disease pressure for CBSD may never, in reality, reach these levels.

II. Secondary infection

Secondary infection, on the other hand, depends upon the distance between a susceptible plant and all infectious plants. Previously research has looked at the effect of a large internal source of CMD infection on a field (Fargette et al., 1990), from which we calculate the effect of a single infected source. We find that an exponential curve

$$I(x) = \beta e^{-\alpha|y-x|},$$

where $\alpha = 0.175$ and $\beta = 0.8952$, best fits an average (both up- and downwind) of the data presented in Fargette et al. (1990), giving R^2=0.8456. $I(x)$ is the incidence of disease at the coordinate x in a one-dimensional plane, y is the coordinate of the source, and the metric $|\cdot|$ measures the distance between the two.

However, as mentioned above, this data describes infection from a large source, while we are interested in the spread of infection from a small (one plant only) source. In order to obtain this from the fitted curve, we determine the density of source plants in the data, and integrate over the transmission model divided by the density; i.e.

$$I(x) = \int_{-\infty}^{\infty} \frac{\beta e^{-\alpha|y-x|}}{\rho(y)} dy,$$

where $\rho(y)$ is the density function of the source at y. In the data, the source consists of 5 rows of plants, with 10 out of 10 plants infected per row, while elsewhere the density of infected plants is assumed to be zero.

Hence we obtain, for a source centred at zero in a one-dimensional plane,

$$I(x) = \frac{\beta}{10}\int_{-2.5}^{2.5} e^{-\alpha|y-x|}dy.$$

From this, for constants of integration p_1 and p_2,

$$I(x) = \begin{cases} \frac{\alpha\beta}{10}\left[e^{-\alpha(x-y)} + p_1\right]_{-2.5}^{2.5}, & \text{if } x > 2.5, \\ \frac{-\alpha\beta}{10}\left[e^{-\alpha(y-x)} + p_2\right]_{-2.5}^{2.5}, & \text{if } x < -2.5. \end{cases}$$

We therefore see that

$$I(x) = \frac{\beta\alpha}{10}e^{-\alpha|x|}\left(e^{2.5\alpha} - e^{-2.5\alpha}\right).$$

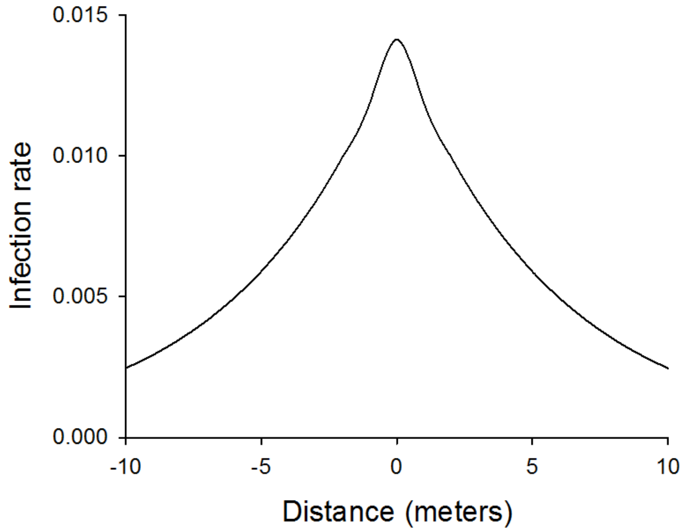

Figure A.1: *CMD infection rate for plants at different distances from a source of infection at zero, estimated from data in Fargette and Vié (1994).*

Note that our secondary infection was calculated at $\tau = 6$ months, when plant susceptibility is greatly reduced (see below) and so we alter β accordingly, such that $\beta = 3.6199$.

In addition, it is convenient to rewrite $\bar{\beta} = \frac{2\pi\beta}{10\alpha d}(e^{2.5\alpha} - e^{-2.5\alpha}) = 0.783$, where $10 \leq d \leq 45$ is the duration in days over which the infection spreads (see the duration of the experiment in Fargette et al., 1990). This may

be thought of as the rate of spread, while $\frac{2}{\alpha}$ is the mean distance of dispersal. We obtain this through noting that the mean distance of dispersal is given by,

$$\int_{-\infty}^{\infty}\int_{-\infty}^{\infty}|y-x|\frac{\alpha^2}{2\pi}e^{-\alpha|y-x|}dxdy,$$

which can be simplified using polar coordinates to obtain

$$\int_{-\infty}^{\infty}\int_{-\infty}^{\infty}|y-x|\frac{\alpha^2}{2\pi}e^{-\alpha|y-x|}dxdy=\frac{\alpha^2}{2\pi}\int_{0}^{\infty}\int_{0}^{2\pi}re^{-\alpha r}rdrd\rho=\frac{\alpha^2}{2\pi}\int_{0}^{2\pi}d\rho\int_{0}^{\infty}r^2e^{-\alpha r}dr=\frac{\alpha^2}{2\pi}2\pi\frac{2}{\alpha^3}=\frac{2}{\alpha}.$$

Combining the above, we obtain infection rates dependent upon distance to source of infection, as demonstrated in Fig. A.1.

The dispersal distance for CBSD is greatly reduced compared to that of CMD (Patil et al., 2014; Legg et al., 2011), so we vary $0.175 \leq \alpha \leq 2$ so that the mean dispersal distance is 1-11.5m.

We presume that the rate of spread for CBSD is similar to that for CMD. Assuming that it is equal to the minimum value for CMD, i.e. $\bar{\beta} = 0.391$, we observe a final incidence for CBSD of between 0 and 10% infection in the field, depending on the initial infection levels. This matches the incidence found in the majority of actual fields (Maruthi et al., 2005; Legg and Raya, 1998). In comparison, choosing the mean value of dispersal from CMD, $\bar{\beta} = 0.783$, results in disease progression to an incidence of over 50%. We therefore choose $\bar{\beta}$ from the minimum of the range for CMD, i.e. $0.391 \leq \bar{\beta} \leq 0.783$. We rescale this to account for plant susceptibility, discussed below, so that $0.406 \leq \bar{\beta} \leq 0.813$.

III. Susceptibility

The rate at which a plant becomes infected also depends on its susceptibility, as mentioned above. Although this is dependent on plant age for CBSD (Hillocks and Jennings, 2003; Rwegasira, 2009), due to a lack of

knowledge of the specifics we must again approximate this based on the dynamics of CMD, outlined in Fargette and Vié (1994, 1995). They assert that susceptibility to CMD depends on plant age τ, and is given by $a(\tau) = 0$ when $\tau \leq 1$ and

$$a(\tau) = 3.01(\tau - 1.07)e^{-1.37(\tau-1.07)} + 0.23$$

when $\tau > 1$, where τ is the plant age in months (see Fig. A.2).

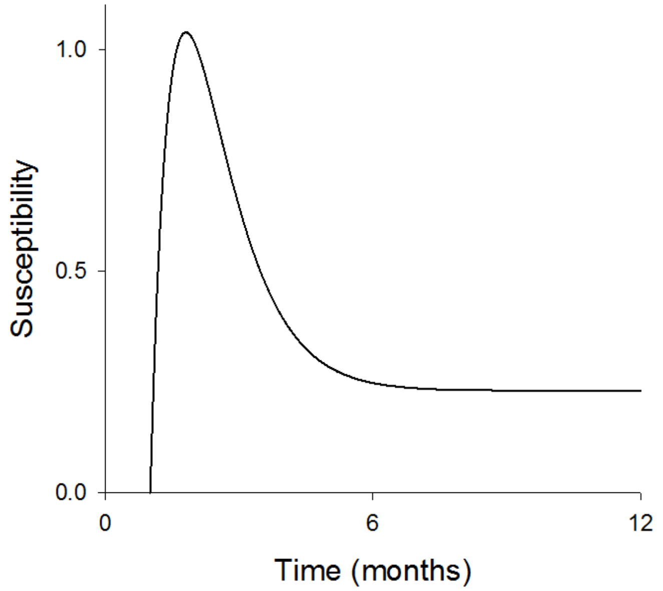

Figure A.2: *Plant age-related susceptibility (Fargette and Vié, 1995).*

IV. Probability of infection

For each plant, we therefore use both primary and secondary infection rates, as well as a rescaling of susceptibility such that it represents a proportion, to calculate the probability of infection in each day (see Box 7.3 in Keeling and Rohani, 2008). In the method outlined in this reference, the two rates of infection, primary and secondary infection, are used to calculate the probability of infection in a given time interval, here considered to be 1 day. To calculate the probability $P(x)$ of a plant at point x in the Cartesian plane becoming infected in one time step Δt, we first consider the probability $e^{-\psi}$ that it is not infected in that time

step, where ψ is the overall rate of infection. Considering also the susceptibility of a plant, and using the metric $||\cdot||$ for the Euclidean distance between plants, the probability of infection for plant x is then given by

$$P(x) = 1 - e^{-a(\tau)z},$$

where

$$z = \frac{\bar{\beta}\alpha^2}{2\pi} \sum_{y \in \text{infected}} e^{-\alpha||y-x||} + \lambda.$$

V. Whitefly dynamics

We then consider the effect of fluctuations in whitefly numbers, which affects both primary and secondary infections. This is modelled through parameter

$$v(t) = v_{\max}\left(-\frac{1}{2}\cos\left(\frac{2\pi t}{365}\right) + \frac{1}{2}\right),$$

for time t in days, where we now define

$$P(x) = 1 - e^{-v(t)a(\tau)z}.$$

This population parameter measures the whitefly present as a proportion of the maximum population, $v_{\max}$, and hence has an amplitude given by $v_{\max}$, as well as a period of 365 days. This is equivalent to a population that reaches a minimum and a maximum once a year, describing the effect of seasonality on whitefly population dynamics (see, for example, Fargette and Vié, 1995; Fargette et al., 1994; Fishpool et al., 1995; Sserubombwe et al., 2001, although see also Legg, 1994). Note that we also define $v(t)$ such that the population peaks 6 months after planting (see Fig. A.3), when the whitefly are most attracted to the foliage of

a cassava crop (Fishpool et al., 1995; Legg, 1994). In addition, although there have been many reports of 200 or more whitefly per plant in some fields (Alicai et al., 2007), here we presume that the field is in an area of lower whitefly density, with a maximum of 110 individuals per plant.

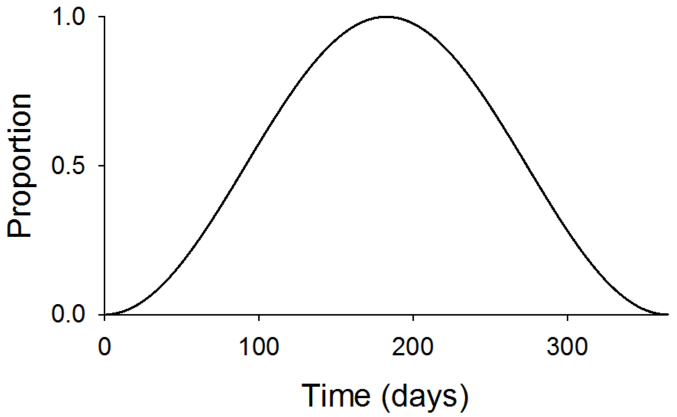

Figure A.3: *Whitefly population dynamics from planting over the growing season.*

VI. Progression and disease identification

The disease becomes symptomatic after $3 - 8$ weeks (Mohammed, 2012, see also Maruthi et al., 2005; Mware et al., 2009), giving an infection progression rate of $g = 0.018 - 0.048$ day^{-1}.

Unexpectedly, it has been observed experimentally that a large proportion of plants may appear symptomatic but are not in fact infected (22%), and importantly for our model, 7% of plants may be infected but are not symptomatic (Rwegasira, 2009; Rwegasira and Rey, 2012). We conclude that the success rate of roguing is essentially unknown, and therefore has a very high range. This is given by $0\% \leq p \leq 93\%$ (see also Abaca et al., 2012), although here we fous on success rates of $50 - 90\%$.

Reversion rates, however, are fairly low, at $5 - 45\%$ (Mohammed, 2012) dependent on cultivar type (Rwegasira and Rey, 2012), and there is little information on cutting selection, so this parameter is taken to be identical to the probability of successful roguing.

Note that all parameters are chosen from uniform distributions over the ranges defined above, which

we collate in table 1 in the text. Parameter estimations are taken from a number of different studies taken at different times at a range of sites across Africa. As such, they do not necessarily accurately represent a geographical area on which this model is focused, but are in many cases the only data available, and as such represent a "best-guess" value for a given parameter.

VII. Elasticity analysis

In Fig. A.4 we conduct an elasticity analysis of the model parameters, given by

$$e_p = \frac{p_{def}}{r_{def}} \cdot \frac{r_{def} - r_{new}}{p_{def} - p_{new}}$$

for each parameter p and result r, where subscript def indicates the default and subscript *new* indicates the value when the parameter is decreased by 5%. We note that roguing frequency and success have an important impact on the incidence of infection, as do both the dispersal and progression rate of the disease.

References

A. Abaca, S.R. Kawuki, P. Tukamuhabwa, Y. Baguma, A. Pariyo, J. Orone, T. Alicai, A. Bua, and C.A. Omongo. Progression of Cassava Brown Streak Disease (CBSD) in infected cassava roots in Uganda. *Uganda Journal of Agricultural Sciences*, 12(1):45–51, 2012.

T. Alicai, C.A. Omongo, M.N. Maruthi, R.J. Hillocks, Y. Baguma, R. Kawuki, A. Bua, G.W. Otim-Nape, and J. Colvin. Re-emergence of cassava brown streak disease in Uganda. *Plant Disease*, 91(1):24–29, 2007.

D. Fargette and K. Vié. Modeling the temporal primary spread of African cassava mosaic virus into plantings. *Phytopathology*, 84(4):378–382, 1994.

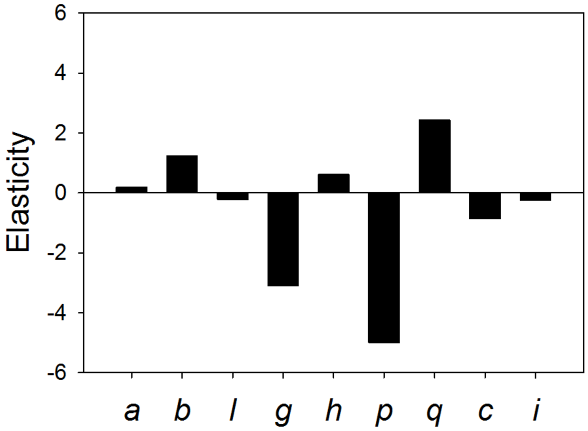

(a) *Incidence, low whitefly*

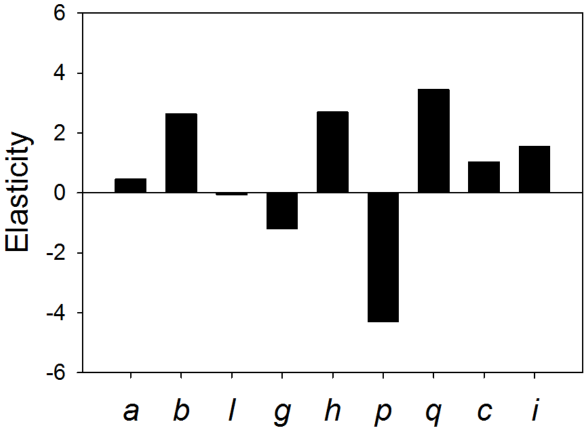

(b) *Incidence, high whitefly*

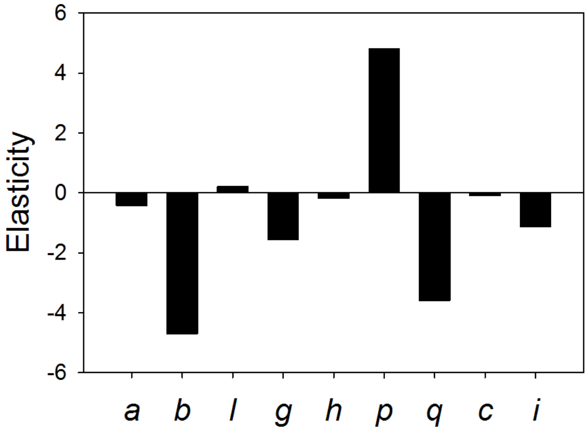

(c) *Cuttings, low whitefly*

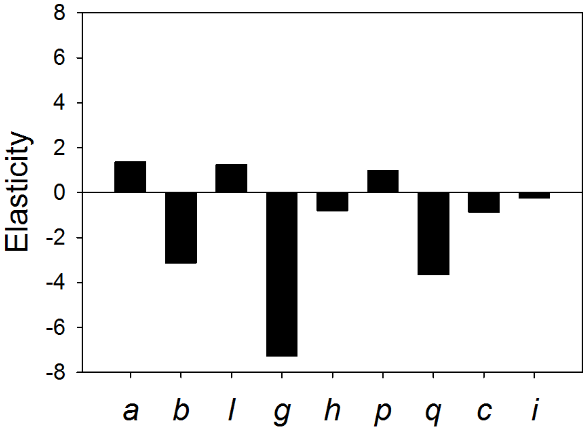

(d) *Cuttings, high whitefly*

Figure A.4: *Elasticity analysis of the model parameters with regards to incidence of infection at harvest in percent (a,b) and percentage of plants available for cuttings (c,d). Whitefly populations are low (35 individuals per plant) in subplots (a) and (c), and high (110 individuals per plant) in subplots (b) and (d). Model parameters are given by $\frac{2}{a}$ (mean dispersal distance), b (rate of spread), l (primary infection rate), g (infection progression rate), h (reversion rate), p (probability of successful roguing), q (roguing frequency), c (probability of successful selection) and i (initial infection).*

D. Fargette and K. Vié. Simulation of the effects of host resistance, reversion and cutting selection on incidence of African cassava mosaic virus and yield losses in cassava. *Phytopathology*, 85(3):370–375, 1995.

D. Fargette, C. Fauquet, E. Grenier, and J.M. Thresh. The spread of African cassava mosaic virus into and within cassava fields. *J. Phytopathology*, 130:289–302, 1990.

D. Fargette, C. Fauquet, and J.M. Thresh. Analysis and modelling of the temporal spread of African cassava mosaic virus and implications for disease control. *African Crop Science Journal*, 2(4):449–458, 1994.

L.D.C. Fishpool, C. Fauquet, D. Fargette, J.-C. Thouvenel, C. Burban, and J. Colvin. The phenology of *bemesia tabaci* (Homoptera: Aleyrodidae) populations on cassava in southern Côte d'Ivoire. *Bulletin of Entomological Research*, 85:197–207, 1995.

R.J. Hillocks and D.L. Jennings. Cassava brown streak disease: a review of present knowledge and research needs. *International Journal of Pest Management*, 49(3):225–234, 2003.

J. Holt, M.J. Jeger, J.M. Thresh, and G.W. Otim-Nape. An epidemiological model incorporating vector population dynamics applied to African cassava mosaic virus disease. *J. App. Ecology*, 34:793–806, 1997.

M.J. Jeger, J. Holt, F. van den Bosch, and L.V. Madden. Epidemiology of insect-transmitted plant viruses: modelling disease dynamics and control interventions. *Physiological Entomology*, 29:291–304, 2004.

M.J. Keeling and P. Rohani. *Modeling Infectious Diseases in Humans and Animals*. Princeton University Press, 2008.

J. Legg and M.D. Raya. Survey of cassava virus diseases in Tanzania. *International Journal of Pest Management*, 44(1):17–23, 1998.

J.P. Legg. *Bemesia tabaci*: the whitefly vector of cassava mosaic geminiviruses in Africa: and ecological perspective. *African Crop Science Journal*, 2(4):437–448, 1994.

J.P. Legg, S.C. Jeremiah, H.M. Obiero, M.N. Maruthi, I. Ndyetabula, G. Okao-Okuja, H. Bouwmeester, S. Bigirimana, W. Tata-Hangy, G. Gashaka, G. Mkamilo, T. Alicai, and P.L. Kumar. Comparing the regional epidemiology of the cassava mosaic and cassava brown streak virus pandemics in Africa. *Virus Research*, 159:161–170, 2011.

M.N. Maruthi, R.J. Hillocks, K. Mtunda, M.D. Raya, M. Muhanna, H. Kiozia, A.R. Rekha, J. Colvin, and J.M. Thresh. Transmission of *cassava brown streak virus* by *bemisia tabaci* (Gennadius). *Journal of Phytopathology*, 153:307–312, 2005.

I.U. Mohammed. *Virus-host interactions in the cassava brown streak disease pathosystem*. PhD thesis, University of Greenwich, 2012.

B.O. Mware, R.D. Narla, R. Amata, F. Olubayo, J.M. Songa, S. Kyamunyua, and E.M. Ateka. Efficiency of cassava brown streak virus transmission by two whitefly species in coastal Kenya. *Journal of General and Molecular Virology*, 1(4):040–045, 2009.

B.L. Patil, J.P. Legg, E. Kanju, and C.M. Fauquet. Cassava brown streak disease: a threat to food security in Africa. *Journal of General Virology*, 2014.

G.M. Rwegasira. *Aspects of the epidemiology of cassava brown streak virus disease in Tanzania*. PhD thesis, University of the Witwatersrand, 2009.

G.M. Rwegasira and C.M.E. Rey. Relationship between symptoms expression and virus detection in cassava brown virus streak-infected plants. *Journal of Agricultural Science*, 4(7):246–253, 2012.

W.S. Sserubombwe, J.M. Thresh, G.W. Otim-Nape, and D.O.S. Osiru. Progress of cassava mosaic virus disease and whitefly populations in single and mixed stands of four cassava varieties grown under epidemic conditions in Uganda. *Ann. appl. Biol.*, 84(1):91–98, 2001.
