# Supplementary material for: Cassava brown streak disease and the sustainability of a clean seed system
Source: Plant Pathol. 2015 Sep 21;65(2):299–309. doi: 10.1111/ppa.12453 (PMC4950135; doi:10.1111/ppa.12453)
Supplement: Supplementary file 2 — Appendix S2. Additional results. [file PPA-65-299-s002.pdf]

Appendix B. Additional results

Fig. B.1 demonstrates the effect of the magnitude of a number of important infection parameters on incidence of disease in a field when the whitefly population is small, in an analogue to Fig. 2 in the main text. Results are qualitatively similar. In addition, in Fig. B.2 we investigate the importance of cutting selection, where diseased cuttings are identified and removed after harvest as a form of final roguing, in order to determine its benefit or otherwise. Note that all other parameters are chosen from the ranges defined in Table 1. Compared to the stochasticity introduced by varying these parameters (dispersal distance and rate, primary infection rate, initial source of infection, disease progression and reversion rates, number of whitefly and roguing intervals), cutting selection makes very little difference to either the final incidence or plant population size in the field.

In Figs. B.3 and B.4 we compare for illustrative purposes the incidence and number of cuttings respectively for a system with monthly roguing at a 70% success rate under different disease pressures. This is achieved through varying the primary infection rate, and is analogous to the middle right hand subplots in Figs. 3 and 4 under different disease pressure. Although disease pressure is often linked to whitefly numbers, we separate the effect of increased whitefly bringing disease into the field, due to historical disease in the surrounding area, from whitefly numbers themselves. We note that as the disease pressure increases the initial infection incidence has less effect. For medium and high disease pressure systems, final incidence increasingly lacks any form of pattern as whitefly numbers increase, due to the plant population density decreasing to very low levels.

Figs. B.5 and B.6 represent expanded results to those in Figs. B.3 and B.4, showing the infection incidence and number of plants available for cuttings after one growing season in a medium pressure system. These are analogous to Fig.s 3 and 4 in the main text, under medium disease pressure. Note that similar results for a high pressure system are essentially random, as the number of plants is negligible (less than 10 % of the initial population for all roguing regimes), and are therefore not included here.

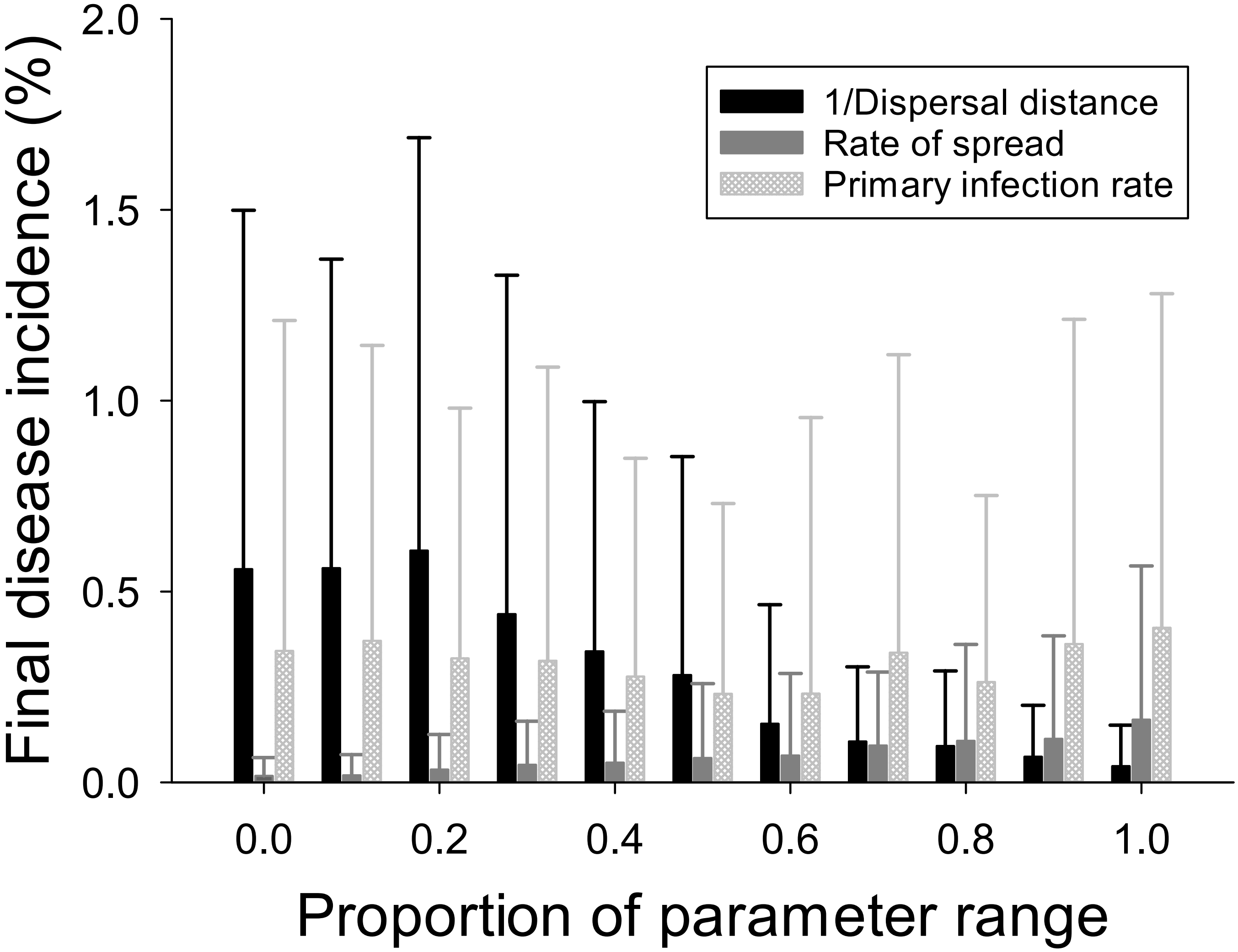

Figure B.1: *Comparisons of the effects of parameter ranges for α, β and λ on final disease incidence in a field with a low (5-35 individuals per plant) whitefly population. The x-axis measures the value for the appropriate parameter as a proportion of the total range found in Table 1. Note that all other parameters are chosen from a uniform distribution given in Table 1. The bar gives the mean and the whiskers one standard deviation for 300 runs of the model.*

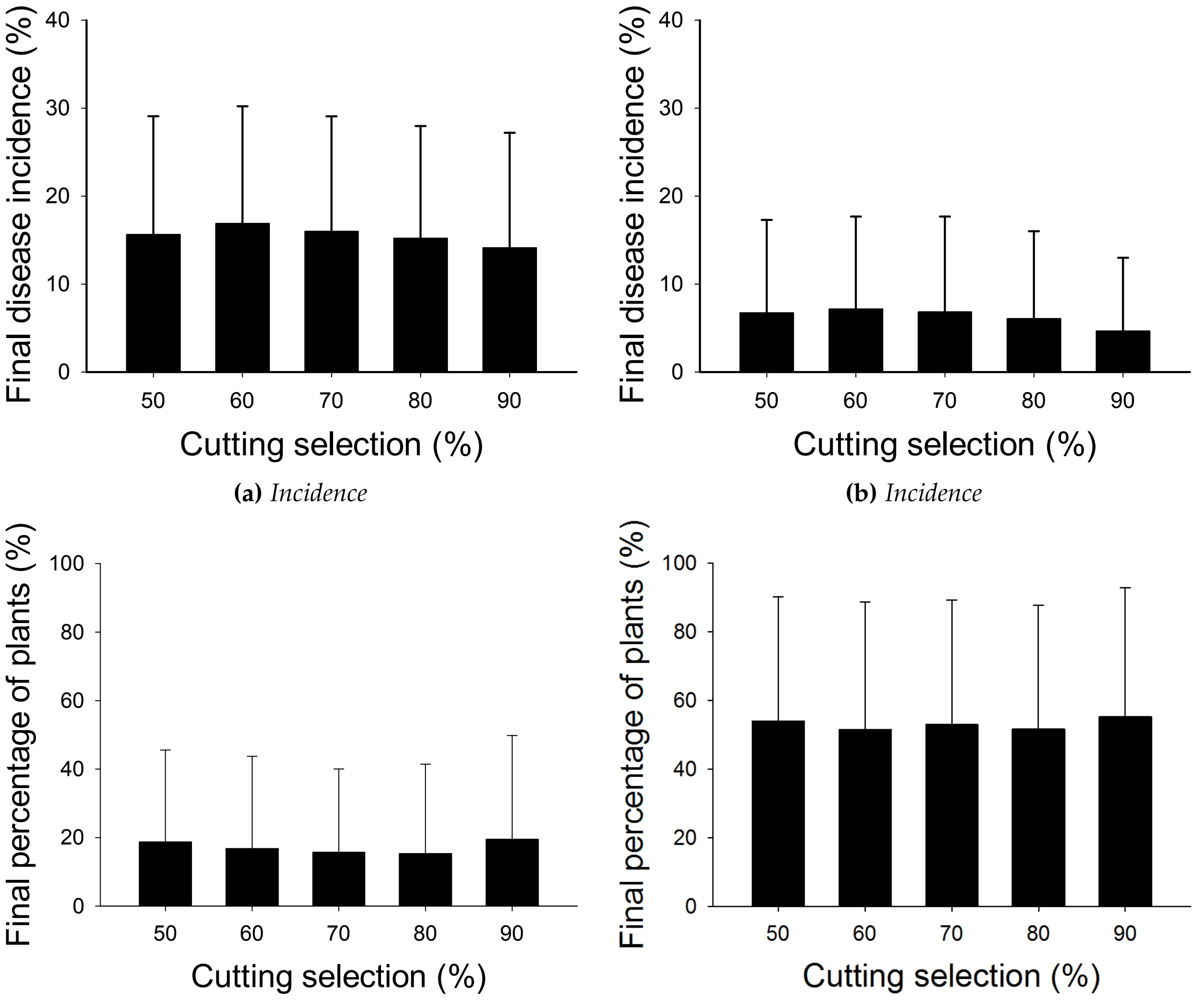

(c) *Population* **(d)** *Population*

Figure B.2: *The effect of variable selection for disease-free harvested cuttings on (a,b) disease incidence levels (%) and (c,d) plant population remaining at harvest (% of the initial population). Whitefly populations are (a,c) high (80-110 individuals per plant) and (b,d) low (5-35 individuals per plant). Roguing success rate is 50%, so cutting selection is taken to be greater than this. The bar gives the mean and the whiskers one standard deviation for 300 runs of the model.*

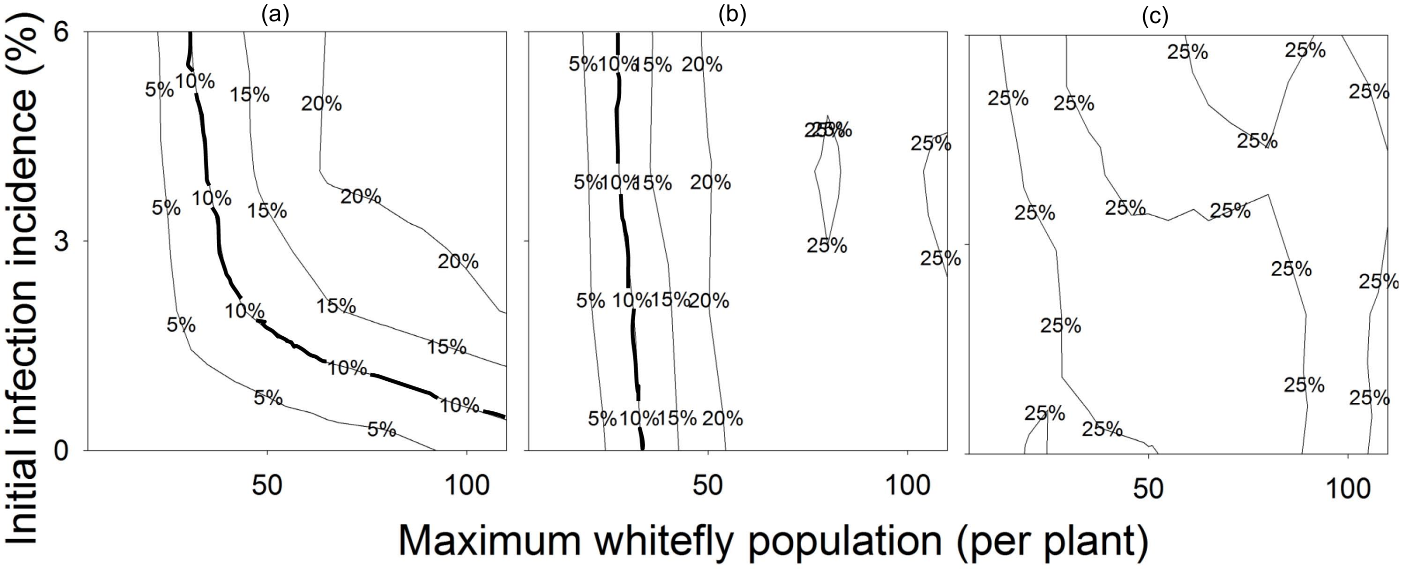

Figure B.3: *Final incidence of disease (%) in cuttings distributed to farmers at harvest. For each plot, x-axes are the maximum whitefly numbers (per plant) and y-axes the size of an initial source of infection (%). Disease pressure varies across columns, and is (a) low ($0 \leq \lambda < 1.8\text{x}10^{-5}$), (b) medium ($1.8\text{x}10^{-5} \leq \lambda < 2.8\text{x}10^{-3}$) and (c) high ($2.8\text{x}10^{-3} \leq \lambda < 2.08$). Bold lines represents the guideline maximum infection level in the cuttings.*

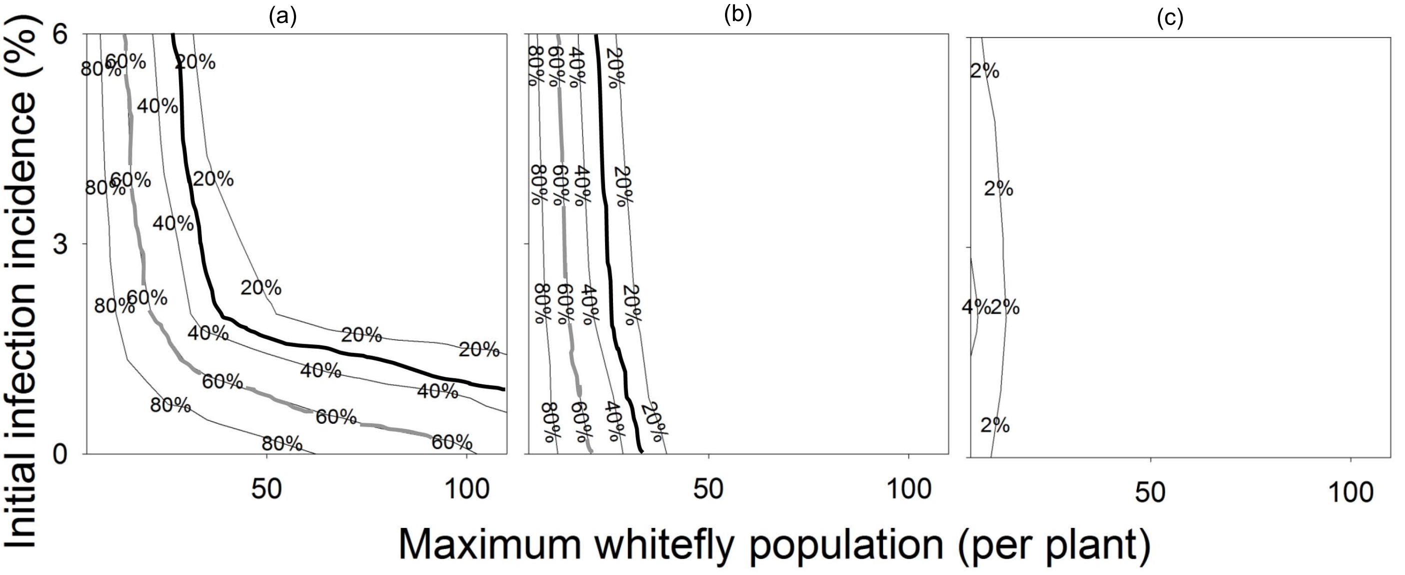

Figure B.4: *Percentage of initial plants available at harvest to provide cuttings to distribute to farmers. For each plot, x-axes are the maximum whitefly numbers (per plant) and y-axes the size of an initial source of infection (%). Disease pressure varies as in Fig. 6. Bold grey lines represents the minimum proportion of plants remaining in a 5 acre field at harvest to distribute sufficient cuttings to plant 30 acres of fields if multiplication rates are high (twenty-fold), while bold black lines represent the same for low multiplication rates (ten-fold)*

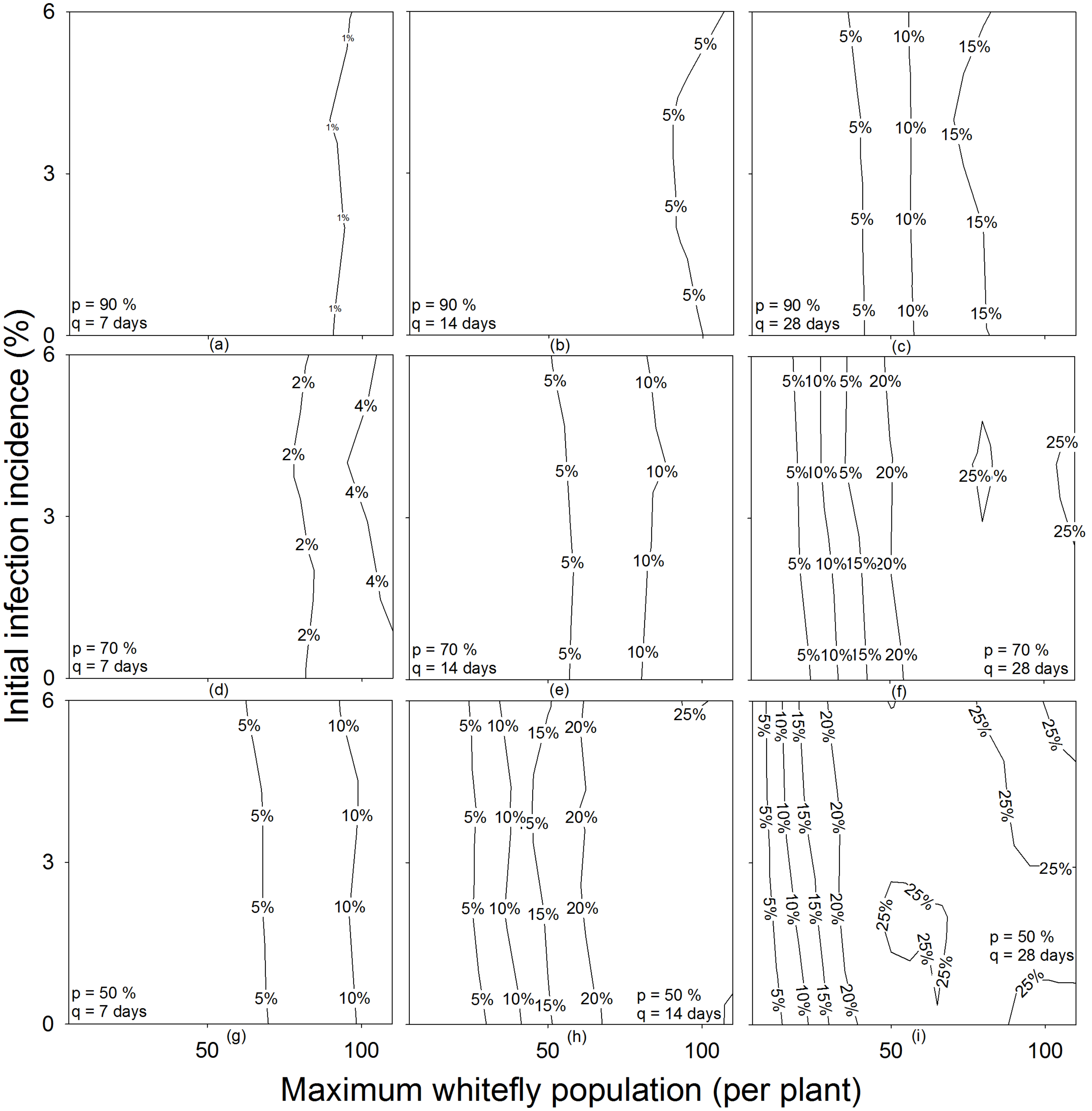

Figure B.5: *Mean final incidence of disease (%) after one season in cuttings distributed to farmers at harvest over a range of parameters for a medium disease pressure system. For each plot, x-axes are the maximum whitefly numbers (per plant) and y-axes the size of an initial source of infection (%). Roguing intervals varies across columns, where the interval given below is for the first two months after planting, after which the interval doubles. Roguing occurs weekly in column 1 (plots a, d, g), fortnightly in column 2 (plots b, e, h) and monthly in column 3 (plots c, f, i). Roguing success, or the probability of successfully detecting a symptomatic plant, varies across rows, and is 90% in row 1 (plots a-c), 70% in row 2 (plots d-f) and 50% in row 3 (plots g-i).*

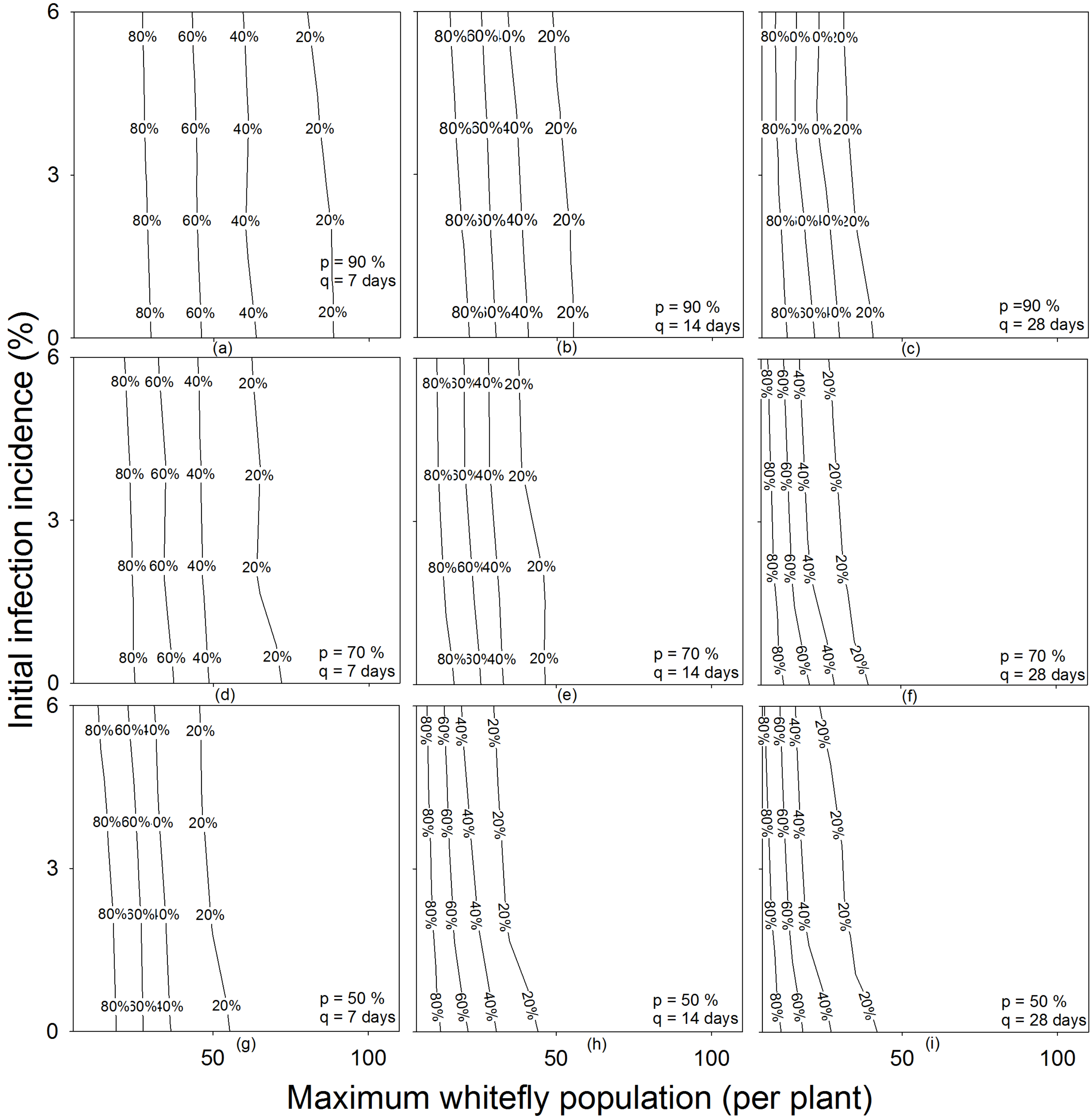

Figure B.6: *Mean number of plants planted available at harvest to provide cuttings (% of the initial plant population) to distribute to farmers, considered over a range of parameters for a medium disease pressure system. X-axes, y-axes, columns and rows vary as described in Fig. B.5.*

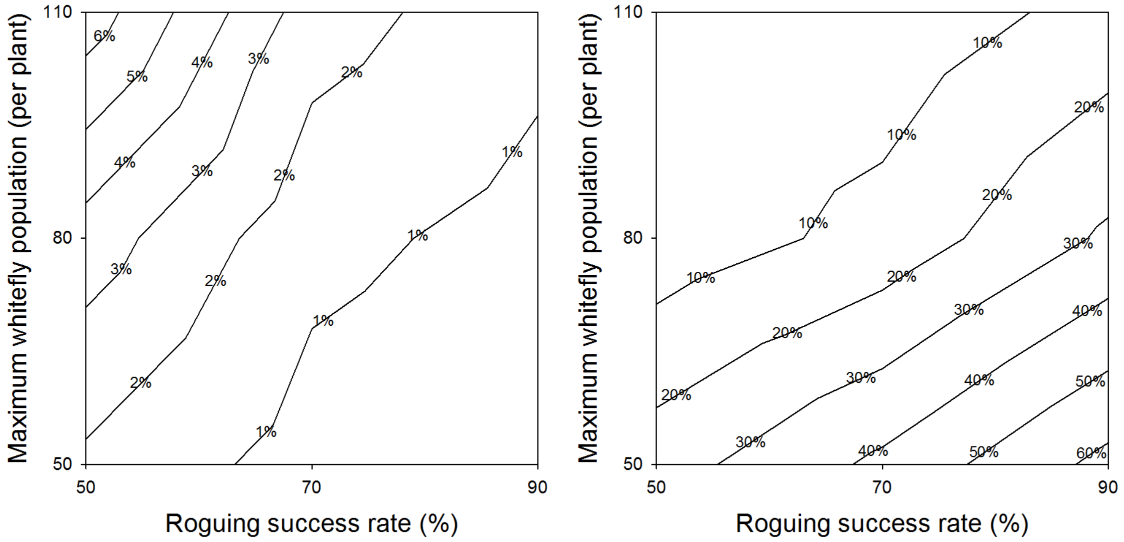

(a) *Incidence* **(b)** *Population*

Figure B.7: *Equilibrium incidence and plant population levels over a ten-year period if roguing is conducted weekly for varying roguing success rates and whitefly populations in a medium-pressure system. Results are for (a) mean final disease incidence (%) and (b) mean number of plants planted available at harvest to provide cuttings (% of the initial plant population).*

Figs. B.7 and B.8 show the degeneration of planting material over 10 years in systems with medium disease pressure rogued weekly and fortnightly respectively, for a range of parameters. We note that both the incidence and populations in these systems quickly equilibrate to the same level irrespective of initial disease incidence (as is seen in systems in low disease pressure areas) but the low proportion of plants available for cuttings at the end of each growing season severely limits their sustainability.

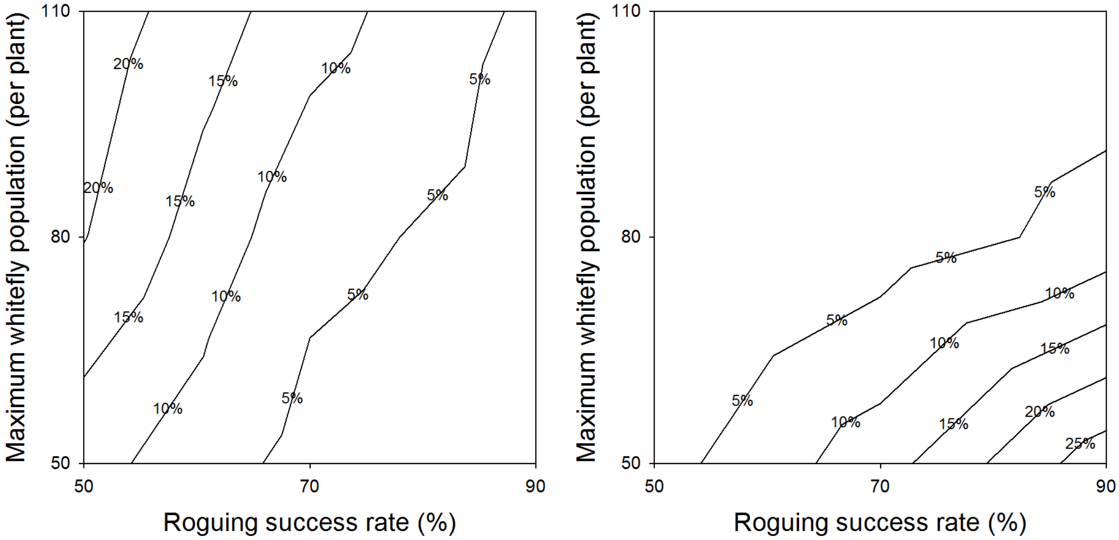

(a) *Incidence* **(b)** *Population*

Figure B.8: *Equilibrium incidence and plant population levels over a ten-year period if roguing is conducted fortnightly for varying roguing success rates and whitefly populations in a medium-pressure system. Results are for (a) mean final disease incidence (%) and (b) mean number of plants planted available at harvest to provide cuttings (% of the initial plant population).*
